# Understanding the Effects of Social Cohesion on Social Wellbeing: A Scoping Review

**DOI:** 10.3389/ijph.2025.1607414

**Published:** 2025-01-30

**Authors:** Sally Fowler Davis, Megan Davies

**Affiliations:** ^1^ Faculty of Health, Medicine and Social Care, School of Allied Health and Social Care, Anglia Ruskin University, Chelmsford, United Kingdom; ^2^ School of Health Sciences, Faculty of Medicine and Health Sciences, University of East Anglia, Norwich, United Kingdom; ^3^ Nursing Science, Department of Public Health, University of Basel, Basel, Switzerland

**Keywords:** social cohesion, scoping review, wellbeing, marginalised communities, social relations

## Abstract

**Objectives:**

To describe objective social wellbeing in relation to social cohesion.

**Methods:**

A literature search that sought to understand the contribution of social cohesion in the community as a means of achieving social wellbeing in the UK, published in the last 10 years.

**Results:**

Social cohesion is widely associated with community assets, trust, and a sense of belonging at neighbourhood level. Segregation of sub-groups and “incivilities” can lead to reduced social connectedness and wellbeing. Wider multicultural engagement over time, may be beneficial for social cohesion. Evidence suggests that sufficient facilitation through facilities and services improve social relations and wellbeing and create more cohesive communities. A particular focus is needed on potential minorities within otherwise cohesive communities.

**Conclusion:**

Social cohesion relates to community resilience and the experience of social connectedness at community level. These features can protect vulnerable groups from exclusion and may have other benefits to health and wellbeing.

## Introduction

Social wellbeing is widely associated with access to community assets including good quality housing, local transport, and health and care services as well as low incidents of crime, and safety [1, 2]. Whilst social wellbeing is most often associated with personal or subjective experiences [3] the literature about subjective social experience is vast and conceptually complex. Therefore this scoping review sought to understand notions of “objective social wellbeing” as fundamental to a healthy society [4] particularly focusing on diversity and intersectionality. An investigation of social cohesion was selected as a core measurable phenomenon.

Social cohesion has three component parts: 1. Social relations, 2. Identification with the geographical “place” and 3. An orientation towards the common good for the community [5]. These elements seek to represent the capacity and capability of a neighbourhood to achieve a communal sense of shared inclusion. In other words, a “collective” or an objective experience of wellbeing [6]. 1. Social relations reflect the presence of social networks; the trust that people experience in their neighbours and within the community; and the acceptance of diversity as a local phenomenon. Addressing “What Works for Wellbeing,” a systematic review in 2017 [7] recognised social relations as an important determinant of individual and community wellbeing. 2. Identification with place involves neighbourhood identity, a level of trust in institutions providing support and perceived fairness/equality within the community. In addition, familiarity and strength of relationships can be more significant than the length of time spent living in a particular area. 3 A focus on the common good concerns the experience of solidarity and helpfulness; the respect for social rules; and the degree of civic participation that can be evidenced [8].

Social cohesion is an important concept that is increasingly cited in policy and strategy as a means of achieving social equity and tackling social and economic inequalities, particularly in health and social care. The three key components of social cohesion are recognised as a direct cause or as a buffer to mitigate adverse social conditions (e.g., poverty or unemployment). Policies, variously labelled “integration,” “cohesion” or “community cohesion,” are commonly seen as presenting a positive image of place, but there is much confusion as to what these mean and how they should be translated into policy and practice [9]. The potential for measurement of the three components of social cohesion attracts policymakers, given the evidence that suggests mental wellbeing and health benefits for populations in more cohesive societies [10].

## Methods

This scoping review was systematically conducted to identify high quality and relevant studies, based on the contemporary concerns related to community and “place.” The scoping review aimed to identify “**what evidence of social cohesion results in population or community wellbeing?**.” The study was undertaken as part of a more extensive investigation sponsored by Carnegie UK who were concerned about collective social wellbeing, inequalities in wellbeing across society in the UK, including potential measures of social wellbeing [6].

The design of the scoping review was based on a five-phase approach that Arskey and O’Malley developed in 2005 [11] that includes, developing a well formulated research question; identifying high quality studies to review; extracting selective and relevant data from documents; and synthesising data in the final review using appropriate techniques (e.g., thematic analysis). According to Arskey and O’Malley the synthesis is a means of explaining and presenting critical and trustworthy dataon which to base further investigations [12]. Searches comprised terms for social cohesion combined with protected characteristics that are considered risk factors to inclusive community social wellbeing (see Supplementary Material). Searches were undertaken in SCOPUS, an academic literature search engine for social sciences and using IDOX [TM], a local authority accessible database for grey literature. Given the breath of literature the title and abstract screening was undertaken by the researchers based on the following selection criteria for inclusion, and exclusion, with a specific focus on social cohesion as a phenomena and specifically excluding material that related to animals, children and experimental methods.

### Inclusion Criteria

• UK peer reviewed publications,
• Reports within the IDOX selection
• Specific reference to increased/decreased wellbeing
• Specific reference to elements of social cohesion i.e., social relations/place affiliations/connectedness at community level/meaningful engagement
• Literature referencing intersectionality and with diversity and inclusion in community social wellbeing
• Higher quality (based on journal impact factor); higher relevance.

### Exclusion Criteria

• Published internationally not in English
• Focused on digital interventions or other specific experimental studies that were controlled for psychological testing of individuals or laboratory based or specific to the impacts of COVID-19
• Specific studies of children or older adults where outcomes were specific to age-related population groups
• Overtly referencing loneliness as a subjective experience of wellbeing
• Papers referring to non-human social cohesion.

## Results

**FIGURE 1:**
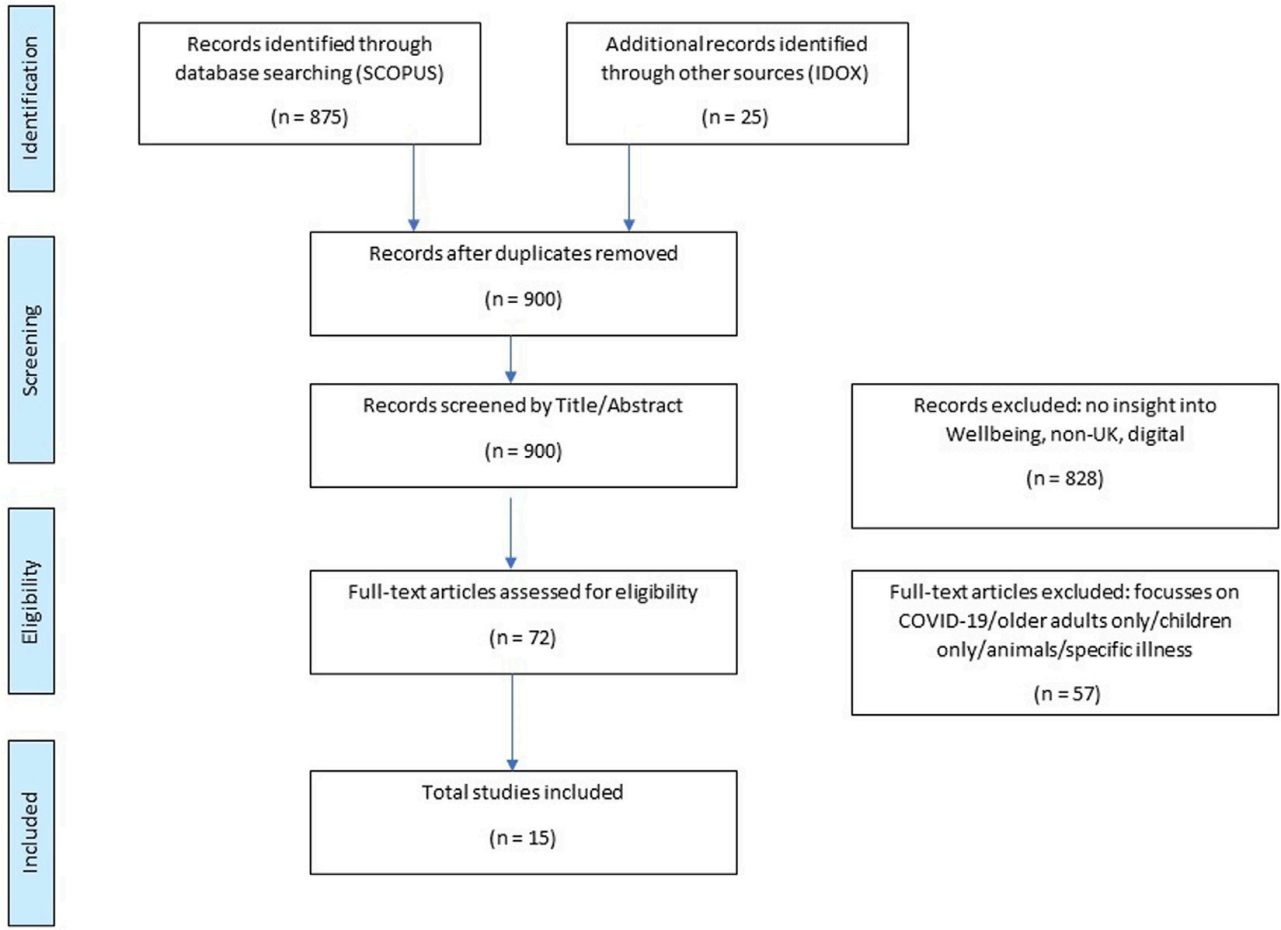
Process of study selection (PRISMA) Identification Screening Eligibility Included (Switzerland 2024).

The outcome of searches is presented below in Figure 1 using a PRISMA diagram to identify how exclusion and selection was undertaken.

The selection 15 papers can be seen in Supplementary Table S1 Selected articles in Supplementary Material. These papers represented a diversity of studies and reports but revealed how social cohesion is being investigated in relation to assets and social capital but also as a risk factor for mental health and population wellbeing. Of note is a finding that higher deprivation doesn’t always lead to low social cohesion, although low social cohesion always negatively impacted mental health [10]. The selected studies are within the three core elements of social cohesion [5].

### Social Relations

Fone et al (2014) in a longitudinal analysis (10) suggests that social cohesion in neighbourhoods has a greater impact on mental health than the neighbourhood being economically deprived. The explanation is that cohesive neighbourhoods demonstrate a tangible increase in support and interaction, therefore mitigating the negative effects of poverty.

Friendships and exchange of favours with neighbours facilitate social networks and improve mental wellness. A sense of trust, a feeling of belonging to the local community and feeling valued appear to be the strongest predictors of both individual and subjective wellbeing. The “exchange of favours” with neighbours facilitates social interaction and may increase levels of social cohesion [13] based on the social bonds that help neighbours achieve the social ties that enable a community to achieve a stable and predictable public environment [14]. Furthermore, people who think they know people within their area and have social connections as well as trust in others will report “good health” significantly more often than those who don’t [15]. Social connectivity appears to differentiate attitudes to wellbeing with “Neighbours who look out for each other” strongly associated with social cohesion (and wellbeing) and conversely “People drunk/rowdy in public” and “Troublesome neighbours” reflecting social disorder and disharmony [16]. Similarly, perceived social/environmental incivilities such as antisocial behaviour, resulted in reduced wellbeing [17]. This is where a higher proportion of older adults (over 65) and higher levels of social housing, worklessness and concentrations of asylum seekers, tend to result in fewer opportunities for socialisation, and contact between communities [18].

### Connections With Place and Person

Public space provides opportunities for people to build social connections with one another, foster pride in the area, relax and reflect. Through community organisation and participation i.e., formal volunteering opportunities, people are able to support their own and others’ resilience, and improve the accessibility, use, look and feel of the public space they share with one another [19]. A fulfilling community life can also impact physical and mental health, which are core determinants of wellbeing [20]. Social wellbeing derived from formal structured and organised activity is social capital, where the acquired benefits associated with local engagement and participation, improve health and life expectancy [21]. Urban spaces designated as “meeting places” are important to build ties and promote reciprocal trust, solidarity, and civic participation e.g., club membership was associated with increased wellbeing for older people [17]. These spaces hold intrinsic value for young people too, providing them with a sense of wellbeing and belonging [22]. Residents suggested that social networks and friendship circles increased where there were purposeful attempts, such as bringing groups of people together for social activities, increasing social connectedness among groups [23]. Further research into the positive effects of green space in particular is warranted, particularly with reference to environmental improvements and urban planning [24].

### Orientation to the Common Good

This aspect of social wellbeing was the least investigated and there is less direct evidence that shared decision making and orientation to better outcomes for all at a local or community level may be a helpful balance to economic factors [25]. A common good could be said to be “better health” and where individual social capital is greatest it is positively associated with better health [26]. This social return on investment in community is specifically linked to a study about not belonging where others do and investigates the impact of social interactions across communities on health and mental wellbeing in Wales. It demonstrates both a positive relationship and mental health outcomes in more cohesive communities but also identifies the risk for marginalised people and sub-groups who are segregated from cohesive communities [26]. Where better health is an accepted “good,” there is a suggestion that social wellbeing outcome tracking can lead to improvements in participants’ outcomes and experience of projects and services. The suggestion here is that it promotes open communication and dialogue with practitioners [27]. The continuous assessment of individual wellbeing and quality of life is both advantageous to a service and to individuals who reflect on their own perceptions of how support is delivered for them.

### Improving Social Cohesion

Socialisation and support at a neighbourhood level have the potential to have a positive impact on social cohesion but also offer a potential mechanism to navigate adverse effects of neighbourhood deprivation [10]. For example, in London, residents suggested that coping with a deprived and hostile environment is balanced with the positive aspects of life on an inner-city estate that include involvement in projects, self-help groups, tenants’ groups as well as courses and toy libraries [20]. Improvements in the economic circumstances of local populations, and investments in resources in local areas such as housing, will minimise perceptions of competition for resources and improve community relations [18].

There was a racial dimension to the definition of social wellbeing that could be defined within inner city, “Black neighbourhoods” where the social capital generated among black youths is both advantageous to social cohesion and wellbeing in the group and somewhat limiting in terms of restricting their activity to a segregated area [22]. Similarly, wellbeing generated within different age groups, such as older adults, was based on specific initiatives to build social relations for the purpose of health and wellbeing improvements [28]. In Scotland, the Community Empowerment Act (2015) [29] is identified as a policy that opened opportunities for “asset-based” work; aiming to empower community bodies to strengthen the communities’ voice in decision making. This “capabilities approach” to wellbeing is central to community development, suggesting that wellbeing is derived from participation and association [30] and residents benefit from new social connections that have been made, particularly with respect to their health and wellbeing.

At a local level, ethnic difference is not a barrier to social wellbeing and cohesion [20] and this contrasts with Putnam’s earlier work suggesting that ethnic diversity reduces social capital [21]. In the long-term, increases in ethnic diversity are likely to promote contact, tolerance and understanding and improve social cohesion [18]. For example, neighbourhood ethnic diversity in London seems to be positively related to the perceived social cohesion of neighbourhood residents [14]. Discourses about how cultural/ethnic/racial identity are promoted are important to address in relation to social wellbeing and multi-culturalism [20]. Additionally, area classification indicates that areas with the lowest levels of social cohesion are those with below average levels of migration and population turnover [18].

Areas with high belonging and apparent cohesion may hide individuals or small groups that experience alienation or isolation, for example, migrant families or older white households within multi-cultural communities. For example, lower physical health-related quality of life is associated with ageing, living in an urban area, and being retired [31]. Where an area is dominated by a particular social group, the health outcomes of the minority may be adversely affected: “where those who do not belong to that group would be keenly aware of the fact” [26]. Risks associated with social wellbeing in low deprivation areas can’t been ignored either, individual or group anxieties and “irritations,” such as gossip among neighbours, can exist and can surface in communities creating “paranoia” [16]. Some of the worst outcomes for small and segregated groups can occur when they are unable to overcome the alienation and segregation they experience. Crime and local incivilities are particularly damaging to social cohesion [18].Community identities and collective narratives are important to establish a shared sense of belonging that help people to feel secure and connected to their community [19] and build resilience to adversity tends to involve notions of friendship, tolerance, and openness [20].

## Discussion

The physical environment and public spaces in particular can be conducive to social cohesion and neighbourliness includes the exchange of favours, leading to trust and reciprocity. Social cohesion may be important to build or maintain local resilience with and social connectedness at a community level insulates more vulnerable groups from isolation and alienation that puts them at risk of poor mental health and low wellbeing. Social cohesion can insulate people from poor mental wellbeing in more deprived areas. However, negative experiences of crime, rowdy behaviours and “paranoia” can reduce the positive effect of social connectedness. Older people experience lower social wellbeing due to physical disability and through marginalisation where the effect of social capital dissipates.

Several papers include reports with empirical testing of wellbeing via the Warwick Edinburgh Mental Wellbeing Scale [15, 17]. However, (as previously stated) this paper does not include a review of methods used to aggregate individual, subjective wellbeing about subjective wellbeing into a measure of social cohesion. The diversity of evidence in the scoping review meant that meta-analysis of the quantitative studies was not been possible.
