# Supplementary material for: Understanding the Effects of Social Cohesion on Social Wellbeing: A Scoping Review
Source: Int J Public Health. 2025 Jan 30;70:1607414. doi: 10.3389/ijph.2025.1607414 (PMC11821421; doi:10.3389/ijph.2025.1607414)
Supplement: Supplementary file 1 [file DataSheet2.PDF]

Table 2: *Selected articles*

Author(s)	Year	Title	Document Type	Methodology	Definition of Wellbeing
Harris, Androulla et al.	2022	How are we doing? A coproduced approach to tracking young Black men's experiences of community wellbeing and mental health programmes	Evaluation of Intervention	Qualitative	Social wellbeing relates to social relatedness and measuring service outcomes for black men in a single community setting
MIND	2022	Together through tough times: the power of community to support mental wellbeing across the UK	Empirical	Qualitative	Subjective community wellbeing as well as individual, via selection of factors that promote social relatedness
McGowan V.J., Akhter N., Halliday E., Popay J., Kasim A., Bambra C.	2022	Collective control, social cohesion and health and well-being: baseline survey results from the communities in control study in England	Empirical	Quantitative Analysis	Social wellbeing as a feature of mental health measured by a shortened version of the Warwick Edinburgh Mental Wellbeing Scale, but also by self-reported 'general health'.
Saville C.W.N.	2021	Not belonging where others do: A cross-sectional analysis of multi-level social capital interactions on health and mental well-being in Wales	Empirical	Analysis	Wellbeing was broken down into 4 questions: ‘Overall, how anxious did you feel yesterday?’, ‘Overall, how happy did you feel yesterday?’, ‘Overall, to what extent do you feel that the things you do in your life are worthwhile?’, and ‘Overall, how satisfied are you with your life nowadays?’
Boyce, Christopher et al.	2020	Understanding Wellbeing	Conceptual	Qualitative analysis	Rigorous measurement of the quality of community life, to determine its correlation to

					wellbeing indicators such as life satisfaction and physical and mental health.
Jones, R., Heim, D., Hunter, S., Ellaway, A.	2020	The relative influence of neighbourhood incivilities, cognitive social capital, club membership and individual characteristics on positive mental health	Empirical	Quantitative analysis	Mental wellbeing is measured using the Warwick Edinburgh Mental Wellbeing Scale. Physical health was self-reported and assessed by adding the total number of illnesses/conditions recorded from a list of 19.
Williams A.J., Maguire K., Morrissey K., Taylor T., Wyatt K.	2020	Social cohesion, mental wellbeing and health-related quality of life among a cohort of social housing residents in Cornwall: A cross sectional study	Empirical	Quantitative Analysis	Social wellbeing relates to physical functioning, role physical, bodily pain, general health, vitality, social functioning, and emotional and mental health / quality of life.
McElroy E., McIntyre J.C., Bentall R.P., Wilson T., Holt K., Kullu C., Nathan R., Kerr A., Panagaki K., McKeown M., Saini P., Gabbay M., Corcoran R.	2019	Mental Health, Deprivation, and the Neighborhood Social Environment: A Network Analysis	Empirical	Mixed Methods	Social wellbeing is defined through mental health, specifically under the following four areas: anxiety, paranoia and auditory verbal hallucinations
Social Marketing Gateway	2018	Building Community: An evaluation of asset based community development (ABCD) in Ayrshire	Evaluation of Intervention	Mixed methods	Asset-based, or ‘positive approaches’, get better outcomes by bringing together people’s assets and skills to develop solutions to community problems.
Mintchev, Nikolay & Moore.	2017	Community and prosperity beyond social capital: the case of Newham, East London,	Conceptual	Qualitative analysis	‘social capital’ is measured by the level of trust that people have in relation to others (social connectivity), as well as the level of

					participation in various organisations and activities (civic engagement).
Fone, D., White, J., Farewell, D., Kelly, M., John, G., Lloyd, K., Williams, G., & Dunstan, F.	2014	Effect of neighbourhood deprivation and social cohesion on mental health inequality: a multilevel population-based longitudinal study	Empirical	Longitudinal analysis	Common mental disorders such as anxiety and depression create a public health burden: Wellbeing is defined by assessment of factors influencing physical and mental health.
Reynolds, T.	2013	Them and us': 'Black neighbourhoods' as a social capital resource among Black youths living in inner-city London	Conceptual	Analysis	Bourdieu's notion of 'habitus' (ways of being) to identify specifically how disadvantaged communities are linked by a ''collective habitus'' 'Black neighbourhoods' in that it allowed him to establish bonds of trust and reciprocal relationships
Giebel, C., Hassan, S., Harvey, G., Devitt, C., Harper, L., Simmill-Binning, C.	2022	Enabling middle-aged and older adults accessing community services to reduce social isolation: Community Connectors	Empirical	Qualitative analysis	Wellbeing related to participation and asset- based approaches to wellbeing
Sturgis, P., Brunton-Smith, I., Kuha, J., & Jackson, J.	2014	Ethnic diversity, segregation and the social cohesion of neighbourhoods in London	Empirical	Quantitative analysis	Main focus on ethnic diversity and social cohesion
Lymperopoulou	2020	Immigration and Ethnic Diversity in England and Wales Examined Through an Area Classification Framework	Empirical	Quantitative analysis	Main focus on ethnic diversity and social cohesion
