# Supplementary material for: Understanding the Effects of Social Cohesion on Social Wellbeing: A Scoping Review
Source: Int J Public Health. 2025 Jan 30;70:1607414. doi: 10.3389/ijph.2025.1607414 (PMC11821421; doi:10.3389/ijph.2025.1607414)
Supplement: Supplementary file 2 [file DataSheet1.PDF]

**Supplementary Materials**

1. Search Terms

( TITLE-ABS-KEY ( ( "Social Cohesion" ) OR ( "Social Connectedness" ) OR ( "Connectedness" ) OR ( "Social Relations" ) OR ( "Sense of Belonging" ) OR ( "orientation towards the common good" ) ) ) AND ( TITLE-ABS-KEY ( ( "Age" ) OR ( "Religion" ) OR ( "Belief" ) OR ( "Sexual Orientation" ) OR ( "Disability" ) OR ( "Sex" ) OR ( "Gender" ) OR ( "Gender Reassignment" ) OR ( "LGBTQ+" ) OR ( "Race" ) OR ( "Ethnicity" ) OR ( "Pregnancy" ) OR ( "Maternity" ) OR ( "Marriage" ) OR ( "Civil Partnership" ) ) ) AND ( LIMIT-TO ( PUBYEAR , 2023 ) OR LIMIT-TO ( PUBYEAR , 2022 ) OR LIMIT-TO ( PUBYEAR , 2021 ) OR LIMIT-TO ( PUBYEAR , 2020 ) OR LIMIT-TO ( PUBYEAR , 2019 ) OR LIMIT-TO ( PUBYEAR , 2018 ) OR LIMIT-TO ( PUBYEAR , 2017 ) OR LIMIT-TO ( PUBYEAR , 2016 ) OR LIMIT-TO ( PUBYEAR , 2015 ) OR LIMIT-TO ( PUBYEAR , 2014 ) ) AND ( LIMIT-TO ( DOCTYPE , "ar" ) ) AND ( LIMIT-TO ( AFFILCOUNTRY , "United Kingdom" ) ) AND ( LIMIT-TO ( SRCTYPE , "j" ) )

In addition, a grey literature search was undertaken via IDOX, the Knowledge Exchange database via the Knowledge Exchange website. This search was similarly orientated and limited to the last 10 years, aiming to identify key reports published by similar organisations to CUK and to ensure that contemporary evidence from field studies and local strategy was also included.
